# Factors Associated With Opting Out of Automated Text and Telephone Messages Among Adult Members of an Integrated Health Care System

**DOI:** 10.1001/jamanetworkopen.2021.3479

**Published:** 2021-03-26

**Authors:** John F. Steiner, Chan Zeng, Angela C. Comer, Jennifer C. Barrow, Jonah N. Langer, David A. Steffen, Claudia A. Steiner

**Affiliations:** 1 Institute for Health Research, Kaiser Permanente Colorado, Aurora

## Abstract

**Importance:**

Health care systems deliver automated text or telephone messages to remind patients of appointments and to provide health information. Patients who receive multiple messages may demonstrate message fatigue by opting out of future messages.

**Objective:**

To assess whether the volume of automated text or interactive voice response (IVR) telephone messages is associated with the likelihood of patients requesting to opt out of future messages.

**Design, Setting, and Participants:**

This retrospective cohort study was conducted at Kaiser Permanente Colorado (KPCO), an integrated health care system. All adult members who received 1 or more automated text or IVR message between October 1, 2018, and September 30, 2019, were included.

**Exposures:**

Receipt of automated text or IVR messages.

**Main Outcomes and Measures:**

Message volume and opt-out rates obtained from messaging systems over 1 year.

**Results:**

Of the 428 242 adults included in this study, 59.7% were women, and 66.5% were White; the mean (SD) age was 52.3 (17.7) years. During the study period, 84.1% received 1 or more text messages (median, 4 messages; interquartile range, 2-8 messages) and 67.8% received 1 or more IVR messages (median, 3 messages; interquartile range, 1-6 messages). A total of 8929 individuals (2.5%) opted out of text messages, and 4392 (1.5%) opted out of IVR messages. In multivariable analyses, individuals who received 10 to 19.9 or 20 or more text messages per year had higher opt-out rates for text messages compared with those who received fewer than 2 messages per year (adjusted odds ratio [aOR]: 10-19.9 vs <2 messages, 1.27 [95% CI, 1.17-1.38]; ≥20 vs <2 messages, 3.58 [95% CI, 3.28-3.91]), whereas opt-out rates increased progressively in association with IVR message volume, with the highest rates among individuals who received 10.0 to 19.9 messages (aOR, 11.11; 95% CI, 9.43-13.08) or 20.0 messages or more (aOR, 49.84; 95% CI, 42.33-58.70). Individuals opting out of text messages were more likely to opt out of IVR messages (aOR, 4.07; 95% CI, 3.65-4.55), and those opting out of IVR messages were more likely to opt out of text messages (aOR, 5.92; 95% CI, 5.29-6.61).

**Conclusions and Relevance:**

In this cohort study among adult members of an integrated health care system, requests to discontinue messages were associated with greater message volume. These findings suggest that, to preserve the benefits of automated outreach, health care systems should use these messages judiciously to reduce message fatigue.

## Key Points

**Question:**

Are patients who receive a high volume of automated text and interactive voice response telephone messages more likely to opt out of future messages than patients who receive fewer messages?

**Findings:**

In this cohort study of 428 242 adults from an integrated health care system, 2.5% of individuals opted out of future text messages and 1.5% opted out of interactive voice response messages. Individuals who received 10 or more text messages or 2 or more interactive voice response telephone calls were significantly more likely to opt out.

**Meaning:**

The findings suggest that a high volume of automated health care messages may be associated with a greater likelihood of patients opting out of future messages and that health care systems should use these messages judiciously to minimize message fatigue.

## Teaser

This cohort study assesses whether a high volume of automated text or interactive voice response telephone messages is associated with requests to opt out of future messages.

## Introduction

Reminding patients of upcoming clinical appointments, laboratory monitoring, and other scheduled services may be associated with improved efficiency and effectiveness of health care. Systematic reviews have shown that reminders are associated with reduced missed primary and specialty care appointments, increased delivery of preventive services, improved prescription refill rates, and enhanced laboratory monitoring.^1,2,3^ Over time, the technology for delivering reminders has largely shifted from live telephone calls and postal reminders to automated text messages and interactive voice response (IVR) telephone reminders to reduce staff burden and costs. These communication channels have also facilitated campaigns to promote healthy behavior or disseminate information to patients within health care systems.^4^ The COVID-19 pandemic has accelerated the reliance of health care systems on virtual communication channels to inform patients about care options and provide guidance about preventive practices. This reliance will likely increase as systems address the backlog of deferred care associated with the pandemic and continue to emphasize virtual care.^5,6^

Health care systems usually implement messaging interventions for a specific clinical purpose but rarely assess the aggregate volume of messages delivered to an individual patient over time. Little research has quantified the cumulative volume of text and IVR messages that patients receive from their health care system or assessed message fatigue, defined as “an aversive motivational state of being exhausted and bored by overexposure to similar redundant messages over a prolonged period of time.”^7^^(p10)^

In the Kaiser Permanente Colorado (KPCO) health system, randomized clinical trials have demonstrated that text and IVR messages can reduce missed primary care appointments, increase medication refills and laboratory test completion, improve child and adult immunization rates, transmit home blood pressure readings, and facilitate obesity counseling.^8,9,10,11,12,13,14,15^ Thus, these communication channels are widely used within the system. In this study, we assessed the total number of text and IVR messages delivered by KPCO to its adult members over a 1-year period and the association between the volume of messages and message fatigue, defined as requests to stop delivery of text or IVR messages.

## Methods

We conducted a retrospective cohort study to assess text and IVR message fatigue in KPCO members. The study cohort consisted of all adult KPCO members who received at least 1 text or IVR message between October 1, 2018, and September 30, 2019. These individuals were included whether or not they had in-person visits during the year because all members could receive text or IVR messages. Individuals with KPCO insurance for part of the year were included for their period of enrollment. We excluded individuals who had requested not to participate in medical record–based research or who had special restrictions on access to health records. The Kaiser Permanente Colorado institutional review board assessed the study administratively and ruled that it was a quality improvement project that did not constitute human participant research or require participant consent. This study followed the Strengthening the Reporting of Observational Studies in Epidemiology (STROBE) reporting guideline.^16^

During 2018 and 2019, KPCO provided health insurance through traditional health maintenance organization, deductible, and coinsurance plans; Medicare; Medicaid; and other payers. The integrated KPCO health care system delivered integrated primary and specialty care to more than 650 000 children and adult members in Colorado. More than 1200 physicians, along with advanced practice nurses and physician assistants, provided primary and specialty care in 29 clinics and 4 hospitals in the Colorado Front Range region. Because KPCO provided both health insurance and clinical care, the system could identify all adult members eligible to receive text and IVR messages regardless of their use of in-person care.

Since 2005, a technical team located in the KPCO research department has developed text and IVR messages for operational and research purposes. Consistent with the practice of other health care systems, KPCO delivers clinical and operational text and IVR communications without requiring informed consent.^17^ By 2019, text messaging had become the dominant communication channel. Each text message allowed recipients to opt out, defined as a request for discontinuation of all future text messages, by reply text. Each IVR message provided recipients the option to opt out of further messages for that specific clinical purpose.

Although most text and IVR messages were delivered by a regional team, Kaiser Permanente’s national program also delivered some text and telephone messages to KPCO members. In addition, KPCO members could communicate with their health care team and receive reminders, updates, clinical information, and other messages through the Kaiser Permanente patient portal.^18^ By 2019, 78.6% of KPCO members were enrolled in the patient portal, and estimated email volume exceeded 6.5 million messages per year.

We excluded email communications from this analysis because members voluntarily established and used their account on the patient portal and because email communications might be less likely to be associated with message fatigue than text or IVR messages delivered without formal consent. We also excluded text or IVR communications from the Kaiser Permanente national program because records of these contacts were not available. Telephone reminder calls from KPCO staff to patients were excluded because they were not systematically captured in the KPCO electronic health record. Thus, text and IVR reminders issued by the KPCO regional team underestimated total member outreach through all communication channels.

### Study Measures

We defined message volume for each cohort member and each communication channel as the total number of messages that KPCO attempted to deliver during the study year. Text and IVR message volumes were assessed separately. We counted a message even if it was not successfully delivered. Each message was categorized as an appointment reminder, a medication refill reminder, a screening reminder, a vaccination reminder, a diagnostic test reminder, or another reason for communication. Messages sent for research studies were excluded because they did not contribute to clinical care. Some individuals in the cohort were also included in 3 large-scale text message campaigns. In March 2019, adults 65 years and older were informed about the accessibility of primary care in Kaiser Permanente. In June and August 2019, members received an anticipatory message encouraging them to seek care from a KPCO facility rather than a non-KPCO site during the upcoming holidays. These text messages were categorized as text campaigns for this analysis.

We collected information about text and IVR messages from the software systems that delivered the messages (Twilio text message software and Voxeo IVR software). Message variables included a project identifier, the type and date of message (text or IVR), the response (delivered, voicemail, no answer, or busy), and the date and time of the message. The system also recorded whether the member had responded to a text message with a request to stop receiving all future text messages and whether a member had responded to an IVR call with a request to stop receiving future IVR messages for that specific indication. We restricted both message-level and member-level analyses to the first message that prompted an opt-out request.

We collected sociodemographic, insurance, clinical, social, and service use variables for each cohort member from the KPCO electronic health record. Sociodemographic variables included age, sex, and self-reported race/ethnicity. Unknown race/ethnicity was included as a separate category in all analyses. Insurance variables included the type of health plan and payer and duration of enrollment in KPCO. Social information was collected about residential instability (number of home addresses in the study year) and financial need (financial assistance with health care costs from KPCO). Clinical variables included the sum of *International Classification of Diseases and Related Health Problems, Tenth Revision* (*ICD-10*) codes for the 32 health conditions that comprise the Quan comorbidity scale^19^ and mental and behavioral health and substance use conditions with the use of an established set of *ICD-10* codes.^20^ Rates of use for primary and specialty outpatient care visits, emergency department visits, and hospitalizations were calculated for visits to KPCO facilities and out-of-system services paid for by KPCO.

### Statistical Analysis

We conducted separate analyses for text and IVR messages. Individuals who received both text and IVR messages were included in both analyses. We conducted 2-sided bivariate comparisons using *t* tests for continuous variables and χ^2^ tests for categorical variables. For members of the cohort who did not opt out of text and IVR messages, we calculated the rate of messages per year as the number of messages attempted, divided by the proportion in the year during which they were enrolled in the health plan. For individuals who opted out, we calculated the rate of messages per year as the number of messages delivered, divided by the proportion in the year that elapsed before the date of their opt-out request. We used multivariable logistic regression models to examine the association between each outcome (text or IVR) and sociodemographic, insurance, social, and clinical factors. We did not include service use variables in these analyses because use could be directly associated with the reminder text or call. The discrimination of logistic regression models was evaluated using the C statistic. All analyses were performed using SAS, version 9.4 (SAS Institute Inc). A 2-sided *P* < .05 was deemed statistically significant.

## Results

### Characteristics of the Participants

Between October 1, 2018, and September 30, 2019, 612 780 adults were KPCO members for all or part of the year. KPCO sent at least 1 text or IVR message to 428 242 of these individuals (69.9%), who comprised the study cohort. Comparison of the characteristics of adult KPCO members who received any text or IVR message with characteristics of those who did not is given in eTable 1 in the Supplement. The median age of cohort members was 53 years (IQR, 38-66 years; mean [SD], 52.3 [17.7] years), and 59.7% were women. Most were White (66.5%) or Hispanic (15.2%) and were covered by deductible or coinsurance insurance plans (38.0%) or Medicare (29.5%).

### Communication Channels and Opt-Out Rates

**Figure 1.**
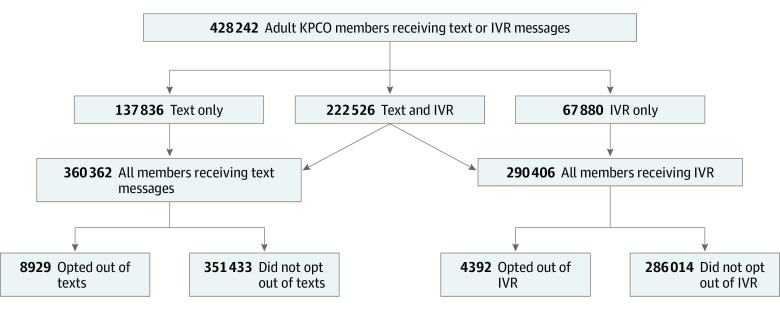
Study Cohort and Analytic Subgroups KPCO indicates Kaiser Permanente Colorado; IVR, interactive voice response.

Figure 1 shows the proportions of cohort members who received no messages, text messages only, IVR messages only, and both text and IVR messages. Overall, 84.1% of cohort members received 1 or more text messages, and 67.8% received 1 or more IVR calls. eTable 2 in the Supplement shows a comparison of characteristics of individuals in the study cohort who received text messages only, IVR calls only, and both types of automated message. Among individuals who received text messages, the median number of messages was 4 (interquartile range [IQR], 2-8). Among those who received IVR messages, the median number of messages was 3 (IQR, 1-6).

During the study year, 8929 individuals (2.5% of cohort members receiving text messages) opted out of at least 1 text message. Among these individuals, 58 (0.6%) opted out a second time after subsequent messages were erroneously sent. During the same period, 4392 cohort members (1.5% of those receiving IVR messages) opted out of at least 1 IVR indication. Among these individuals, 185 (4.2%) subsequently opted out of additional IVR indications. Table 1 shows the sociodemographic, insurance, social, and clinical characteristics and service use among individuals who opted out of 1 or more messages in each communication channel with those among individuals who did not. Compared with participants who did not opt out of text messages, those who did opt out were older (25.2% aged 65-79 years vs 18.9%), more likely to be White (67.9% vs 66.0%), and had more medical conditions (39.7% with 3 or more conditions vs 38.5%) or mental health conditions (11.4% with 3 or more conditions vs 10.3%). Compared with participants who did not opt out of IVR messages, those who did opt out were older (44.9% aged 65-79 years vs 26.5%), more likely to be White (79.8% vs 69.5%), and had more medical conditions (67.6% with 3 or more conditions vs 48.0%) and mental health conditions (19.2% with 3 or more conditions vs 11.8%). Among participants who opted out of text messages, 19.3% had received 20 or more messages, compared with 6.4% of those who did not opt out of text messages. Among individuals who opted out of IVR messages, 33.5% had received 20 or more messages compared with 3.8% of those who did not opt out of IVR messages.

**Table 1.** Characteristics of Adult Members of Kaiser Permanente Colorado Who Opted Out Vs Those Who Did Not Opt Out of Text and IVR Messages

Characteristic	Text messages				IVR message			
	All members who received text messages (n = 360 362)	Did not opt out (n = 351 433)	Opted out (n = 8929)	*P* value	All members who received IVR messages (n = 290 406)	Did not opt out (n = 286 014)	Opted out (n = 4392)	*P* value
**Sociodemographic**								
Age, y								
18-34	81 494 (22.6)	79 740 (22.7)	1754 (19.6)	<.001	41 788 (14.4)	41 468 (14.5)	320 (7.3)	<.001
35-54	123 576 (34.3)	121 248 (34.5)	2328 (26.1)		86 112 (29.7)	85 518 (29.9)	594 (13.5)	
55-64	73 562 (20.4)	71 388 (20.3)	2174 (24.3)		62 951 (21.7)	62 284 (21.8)	667 (15.2)	
65-79	68 744 (19.1)	66 494 (18.9)	2250 (25.2)		77 640 (26.7)	75 666 (26.5)	1974 (44.9)	
≥80	12 986 (3.6)	12 563 (3.6)	423 (4.7)		21 915 (7.5)	21 078 (7.4)	837 (19.1)	
Sex								
Female	214 430 (59.5)	208 768 (59.4)	5662 (63.4)	<.001	179 224 (61.7)	176 455 (61.7)	2769 (63.0)	.07
Male	145 932 (40.5)	142 665 (40.6)	3267 (36.6)		111 182 (38.3)	109 559 (38.3)	1623 (37.0)	
Race/ethnicity								
White	237 871 (66.0)	231 809 (66.0)	6062 (67.9)	<.001	202 333 (69.7)	198 827 (69.5)	3506 (79.8)	<.001
Black	16 417 (4.6)	16 125 (4.6)	292 (3.3)		12 947 (4.5)	12 822 (4.5)	125 (2.8)	
Latinx	57 347 (15.9)	56 187 (16.0)	1160 (13.0)		40 615 (14.0)	40 190 (14.1)	425 (9.7)	
Asian	12 762 (3.5)	12 552 (3.6)	210 (2.4)		9308 (3.2)	9208 (3.2)	100 (2.3)	
Native American	2630 (0.7)	2568 (0.7)	62 (0.7)		2175 (0.7)	2145 (0.7)	30 (0.7)	
Other	11 512 (3.2)	11 199 (3.2)	313 (3.5)		8649 (3.0)	8546 (3.0)	103 (2.3)	
Unknown	21 823 (6.1)	20 993 (6.0)	830 (9.3)		14 379 (5.0)	14 276 (5.0)	103 (2.3)	
Insurance payer								
Deductible or coinsurance	146 532 (40.7)	143 124 (40.7)	3408 (38.2)	<.001	94 853 (32.7)	94 075 (32.9)	778 (17.7)	<.001
High deductible	47 357 (13.1)	45 979 (13.1)	1378 (15.4)		28 765 (9.9)	28 559 (10.0)	206 (4.7)	
Traditional HMO	49 279 (13.7)	48 507 (13.8)	772 (8.6)		37 911 (13.1)	37 623 (13.2)	288 (6.6)	
Medicaid	13 734 (3.8)	13 468 (3.8)	266 (3.0)		10 444 (3.6)	10 368 (3.6)	76 (1.7)	
Medicare	89 318 (24.8)	86 413 (24.6)	2905 (32.5)		107 178 (36.9)	104 247 (36.4)	2931 (66.7)	
Other	14 142 (3.9)	13 942 (4.0)	200 (2.2)		11 255 (3.9)	11 142 (3.9)	113 (2.6)	
Duration of enrollment, y								
≤1	42 218 (11.7)	41 054 (11.7)	1164 (13.0)	<.001	28 021 (9.6)	27 776 (9.7)	245 (5.6)	<.001
2-5	130 564 (36.2)	127 444 (36.3)	3120 (34.9)		86 720 (29.9)	85 800 (30.0)	920 (20.9)	
>5	187 580 (52.1)	182 935 (52.1)	4645 (52.0)		175 665 (60.5)	172 438 (60.3)	3227 (73.5)	
Address changes in prior year								
0	307 375 (85.3)	299 861 (85.3)	7514 (84.2)	.002	253 260 (87.2)	249 334 (87.2)	3926 (89.4)	<.001
1	43 374 (12.0)	42 239 (12.0)	1135 (12.7)		30 108 (10.4)	29 719 (10.4)	389 (8.9)	
≥2	9613 (2.7)	9333 (2.7)	280 (3.1)		7038 (2.4)	6961 (2.4)	77 (1.8)	
Received medical financial assistance								
No	348 546 (96.7)	339 899 (96.7)	8647 (96.8)	.52	278 330 (95.8)	274 208 (95.9)	4122 (93.9)	<.001
Yes	11 816 (3.3)	11 534 (3.3)	282 (3.2)		12 076 (4.2)	11 806 (4.1)	270 (6.1)	
**Clinical**								
Medical conditions								
0	85 894 (23.8)	83 653 (23.8)	2241 (25.1)	<.001	50 778 (17.5)	50 456 (17.6)	322 (7.3)	<.001
1	77 934 (21.6)	76 158 (21.7)	1776 (19.9)		54 298 (18.7)	53 745 (18.8)	553 (12.6)	
2	57 786 (16.0)	56 422 (16.1)	1364 (15.3)		44 957 (15.5)	44 409 (15.5)	548 (12.5)	
≥3	138 748 (38.5)	135 200 (38.5)	3548 (39.7)		140 373 (48.3)	137 404 (48.0)	2969 (67.6)	
Mental or behavioral health conditions								
0	253 308 (70.3)	247 129 (70.3)	6179 (69.2)	<.001	194 882 (67.1)	192 410 (67.3)	2472 (56.3)	<.001
1	35 141 (9.8)	34 329 (9.8)	812 (9.1)		29 500 (10.2)	28 987 (10.1)	513 (11.7)	
2	34 584 (9.6)	33 668 (9.6)	916 (10.3)		31 290 (10.8)	30 726 (10.7)	564 (12.8)	
≥3	37 329 (10.4)	36 307 (10.3)	1022 (11.4)		34 734 (12.0)	33 891 (11.8)	843 (19.2)	
Substance use								
No	350 876 (97.4)	342 222 (97.4)	8654 (96.9)	.007	281 764 (97.0)	277 557 (97.0)	4207 (95.8)	<.001
Yes	9486 (2.6)	9211 (2.6)	275 (3.1)		8642 (3.0)	8457 (3.0)	185 (4.2)	
**Service use**								
Primary care visits in prior year								
0	83 959 (23.3)	81 339 (23.1)	2620 (29.3)	<.001	53 135 (18.3)	52 859 (18.5)	276 (6.3)	<.001
1	111 061 (30.8)	108 640 (30.9)	2421 (27.1)		83 580 (28.8)	82 537 (28.9)	1043 (23.7)	
2	69 843 (19.4)	68 328 (19.4)	1515 (17.0)		58 883 (20.3)	57 992 (20.3)	891 (20.3)	
≥3	95 499 (26.5)	93 126 (26.5)	2373 (26.6)		94 808 (32.6)	92 626 (32.4)	2182 (49.7)	
Specialty care visits in prior year								
0	172 202 (47.8)	167 725 (47.7)	4477 (50.1)	<.001	90 756 (31.3)	89 833 (31.4)	923 (21.0)	<.001
1	72 846 (20.2)	71 294 (20.3)	1552 (17.4)		69 600 (24.0)	68 784 (24.0)	816 (18.6)	
2	38 937 (10.8)	38 114 (10.8)	823 (9.2)		41 289 (14.2)	40 708 (14.2)	581 (13.2)	
≥3	76 377 (21.2)	74 300 (21.1)	2077 (23.3)		88 761 (30.6)	86 689 (30.3)	2072 (47.2)	
ED visits in prior year								
0	312 922 (86.8)	305 260 (86.9)	7662 (85.8)	.002	246 474 (84.9)	242 969 (85.0)	3505 (79.8)	<.001
1	30 877 (8.6)	30 085 (8.6)	792 (8.9)		28 380 (9.8)	27 798 (9.7)	582 (13.3)	
≥2	16 563 (4.6)	16 088 (4.6)	475 (5.3)		15 552 (5.4)	15 247 (5.3)	305 (6.9)	
Hospitalizations in prior y								
0	339 313 (94.2)	331 033 (94.2)	8280 (92.7)	<.001	268 364 (92.4)	264 538 (92.5)	3826 (87.1)	<.001
1	15 631 (4.3)	15 175 (4.3)	456 (5.1)		15 931 (5.5)	15 527 (5.4)	404 (9.2)	
≥2	5418 (1.5)	5225 (1.5)	193 (2.2)		6111 (2.1)	5949 (2.1)	162 (3.7)	
Text messages per y								
0	NA	NA	NA	<.001	67 880 (23.4)	66 051 (23.1)	1829 (41.6)	<.001
1.0-1.9	41 114 (11.4)	40 065 (11.4)	1049 (11.7)		16 240 (5.6)	16 095 (5.6)	145 (3.3)	
2.0-2.9	55 938 (15.5)	54 926 (15.6)	1012 (11.3)		16 646 (5.7)	16 515 (5.8)	131 (3.0)	
3.0-4.9	74 460 (20.7)	73 129 (20.8)	1331 (14.9)		40 495 (13.9)	40 207 (14.1)	288 (6.6)	
5.0-9.9	103 322 (28.7)	101 215 (28.8)	2107 (23.6)		75 658 (26.1)	75 017 (26.2)	641 (14.6)	
10.0-19.9	61 238 (17.0)	59 527 (16.9)	1711 (19.2)		52 161 (18.0)	51 411 (18.0)	750 (17.1)	
≥20	24 290 (6.7)	22 571 (6.4)	1719 (19.3)		21 326 (7.3)	20 718 (7.2)	608 (13.8)	
IVR messages per y								
0	137 836 (38.2)	135 239 (38.5)	2597 (29.1)	<.001	NA	NA	NA	<.001
1.0-1.9	63 322 (17.6)	62 159 (17.7)	1163 (13.0)		75 864 (26.1)	75 682 (26.5)	182 (4.1)	
2.0-2.9	39 903 (11.1)	39 058 (11.1)	845 (9.5)		49 903 (17.2)	49 707 (17.4)	196 (4.5)	
3.0-4.9	45 327 (12.6)	44 067 (12.5)	1260 (14.1)		58 143 (20.0)	57 673 (20.2)	470 (10.7)	
5.0-9.9	44 165 (12.3)	42 579 (12.1)	1586 (17.8)		61 722 (21.3)	60 702 (21.2)	1020 (23.2)	
10.0-19.9	21 566 (6.0)	20 598 (5.9)	968 (10.8)		32 294 (11.1)	31 242 (10.9)	1052 (24.0)	
≥20	8243 (2.3)	7733 (2.2)	510 (5.7)		12 480 (4.3)	11 008 (3.8)	1472 (33.5)	
Opted out of IVR message								
No	357 799 (99.3)	349 290 (99.4)	8509 (95.3)	<.001	NA	NA	NA	NA
Yes	2563 (0.7)	2143 (0.6)	420 (4.7)		NA	NA	NA	NA
Opted out of text message								
No	NA	NA	NA	NA	284 074 (97.8)	280 102 (97.9)	3972 (90.4)	<.001
Yes	NA	NA	NA	NA	6332 (2.2)	5912 (2.1)	420 (9.6)	

Abbreviations: ED, emergency department; HMO, health maintenance organization; IVR, interactive voice response (telephone); NA, not applicable.

### Characteristics of the Text and IVR Messages

**Figure 2.**
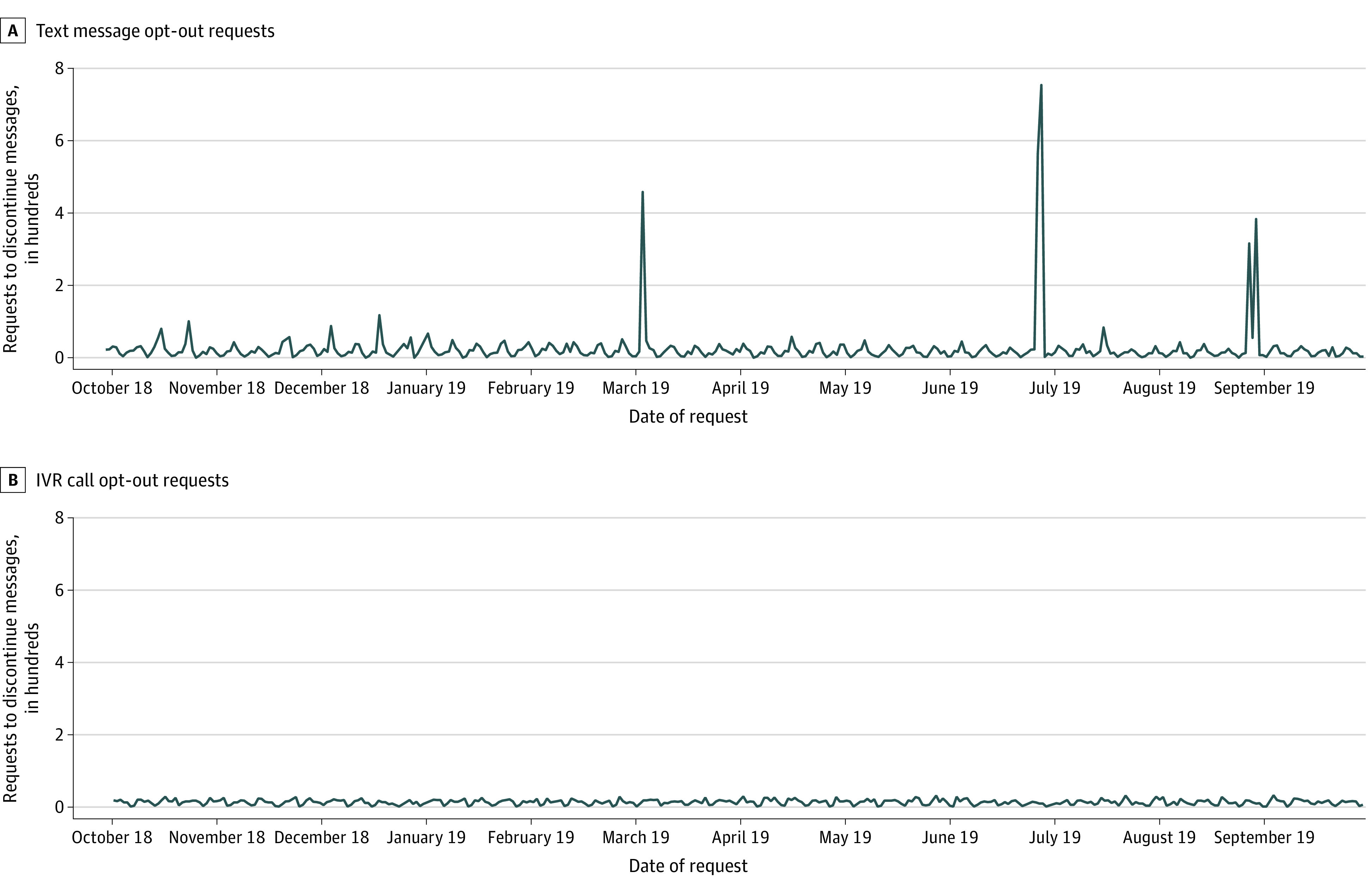
Requests to Opt Out of Automated Text and Interactive Voice Response Messages by Week From October 1, 2018, to September 30, 2019 Three spikes in requests followed messages delivered to large numbers of Kaiser Permanente Colorado members.

Table 2 shows the characteristics of the 3 647 777 messages delivered to participants in the study cohort. Of these messages, 62.2% were delivered by text. Appointment reminders constituted 78.1% of text messages and 91.5% of IVR messages. The opt-out rate for text messages (3.93 per 1000 messages) was significantly higher than that for IVR calls (3.15 per 1000 messages) (rate ratio, 1.25; 95% CI, 1.20-1.30). Compared with opt-out rates for appointment reminders, opt-out rates were significantly higher for most other common text message types, whereas opt-out rates were comparable for most IVR message types. During the 1-year period, spikes in requests to discontinue text messages occurred within 48 hours of the 3 informational text message campaigns, and the discontinuation rate within 48 hours of the 3 informational text message campaigns was 19.11 per 1000 messages (95% CI, 18.36-19.89 per 1000 messages) (Figure 2). Otherwise, little seasonal variation was evident for either communication channel.

**Table 2.** Automated Text and Telephone Messages Among Adult Members of Kaiser Permanente Colorado From October 1, 2018, to September 30, 2019

Type of message	Total messages, No. (%)	Patients who received 1 or more messages^a^	Opt outs after message, No. (%)	Rate of opt outs, No. per 1000 messages sent (95% CI)	Rate ratio (95% CI)
Text messages	2 271 760	360 362	8929	3.93 (3.85-4.01)^b^	NA
Appointments	1 774 457 (78.1)	297 192 (82.5)	3632 (40.7)	2.05 (1.98-2.11)	1 [Reference]
Diagnostic tests	6040 (0.3)	3103 (0.9)	42 (0.5)	6.95 (5.14-9.41)	3.40 (2.51-4.61)
Medication refills	214 755 (9.5)	83 622 (23.2)	914 (10.2)	4.26 (3.99-4.54)	2.08 (1.93-2.24)
Screening	138 516 (6.1)	109 946 (30.5)	1865 (20.9)	13.46 (12.87-14.09)	6.58 (6.22-6.96)
Vaccinations	5651 (0.2)	3219 (0.9)	44 (0.5)	7.79 (5.79-10.46)	3.80 (2.83-5.12)
Text campaigns	126 691 (5.6)	77 229 (21.4)	2421 (27.1)	19.11 (18.36-19.89)	9.34 (8.87-9.83)
Other	5650 (0.2)	4942 (1.4)	11 (0.1)	1.95 (1.08-3.52)	0.95 (0.53-1.72)
IVR messages	1 395 787	290 406	4392	3.15 (3.06-3.24)	NA
Appointments	1 276 608 (91.5)	263 369 (90.7)	4202 (95.7)	3.29 (3.19-3.39)	1 [Reference]
Diagnostic tests	3965 (0.3)	1980 (0.7)	17 (0.4)	4.29 (2.67-6.90)	1.30 (0.81-2.10)
Medication refills	49 065 (3.5)	25 256 (8.7)	139 (3.2)	2.83 (2.40-3.35)	0.86 (0.73-1.02)
Screening	59 548 (4.3)	55 530 (19.1)	15 (0.3)	0.25 (0.15-0.42	0.08 (0.05-0.13)
Vaccinations	2506 (0.2)	1596 (0.5)	4 (0.1)	1.60 (0.60-4.25)	0.48 (0.18-1.29)
Other	4095 (0.3)	3623 (1.2)	15 (0.4)	3.66 (2.21-6.08)	1.11 (0.67-1.85)
Total	3 647 777	428 242	13 321	3.65 (3.59-3.71)	NA

Abbreviations: IVR, interactive voice response (telephone); NA, not applicable.

^a^ Percentages do not add to 100% because individuals may have received multiple types of messages.

^b^ The opt-out rate for text messages was significantly higher than for IVR calls (rate ratio 1.25, 95% CI, 1.20-13.0).

### Characteristics Associated With Message Fatigue

Table 3 shows the bivariate and multivariate associations between member characteristics and opting out of either text or IVR messages. After multivariable adjustment, age 65-79 (aOR, 1.59; 95% CI, 1.40-1.81) and Medicare insurance (aOR, 1.49; 95% CI, 1.31-1.70) were associated with a higher rate of requests to opt out of text messages, whereas receipt of financial assistance for health care costs (aOR, 0.68; 95% CI, 0.60-0.77) and 3 or more medical conditions (aOR, 0.66; 95% CI, 0.61-0.71) were associated with a lower rate of opt-out requests. These relationships were also present for IVR messages: age 65-79 (aOR, 2.17; 95% CI, 2.19-3.36), Medicare insurance (aOR, 1.37; 95% CI, 1.13-1.67), medical financial assistance (aOR, 0.71; 95% CI, 0.63-0.81), and 3 or more medical conditions (aOR, 0.81; 95% CI, 0.71-0.93). In multivariable analyses, individuals who received 10 to 19.9 or 20 or more text messages per year had higher opt-out rates for text messages compared with those who received fewer than 2 messages per year (adjusted odds ratio [aOR]: 10-19.9 vs <2 messages, 1.27 [95% CI, 1.17-1.38]; ≥20 vs <2 messages, 3.58 [95% CI, 3.28-3.91]), whereas opt-out rates increased progressively in association with IVR message volume, with the highest rates among individuals who received 10.0 to 19.9 messages (aOR, 11.11; 95% CI, 9.43-13.08) or 20.0 messages or more (aOR, 49.84; 95% CI, 42.33-58.70). This pattern was most pronounced among the 4.3% of cohort members who received 20 or more IVR calls per year compared with those who received fewer than 2 IVR calls per year (adjusted odds ratio [aOR], 49.8; 95% CI, 42.3-58.7). Individuals who opted out of receiving text messages were also more likely to opt out of receiving IVR messages (aOR, 4.07; 95% CI, 3.65-4.55), and individuals who opted out of receiving IVR calls were also more likely to opt out of receiving text messages (aOR, 5.92; 95% CI, 5.29-6.61) . Although the same variables were included in both models, the model for text messages had substantially lower discrimination (C statistic, 0.66; 95% CI, 0.65-0.67) than the model for IVR messages (C statistic, 0.83; 95% CI, 0.82-0.84). Multivariable models limited to active users of the health care system who had 1 or more in-person visits during the study year showed comparable associations in multivariable models (eTable 3 in the Supplement).

**Table 3.** Bivariate and Multivariate Analyses of Opting Out of Text and IVR Message

Characteristic	OR (95% CI)			
	Opted out of text message		Opted out of IVR message	
	Unadjusted	Adjusted	Unadjusted	Adjusted
**Sociodemographic**				
Age, y				
18-34	1 [Reference]	1 [Reference]	1 [Reference]	1 [Reference]
35-54	0.87 (0.82-0.93)	0.97 (0.91-1.03)	0.90 (0.79-1.03)	0.98 (0.85-1.13)
55-64	1.38 (1.30-1.48)	1.53 (1.42-1.64)	1.39 (1.21-1.59)	1.34 (1.16-1.55)
65-79	1.54 (1.44-1.64)	1.59 (1.40-1.81)	3.38 (3.00-3.81)	2.17 (1.77-2.66)
≥80	1.53 (1.37-1.70)	1.59 (1.35-1.86)	5.15 (4.52-5.86)	2.71 (2.19-3.36)
Sex				
Male	1 [Reference]	1 [Reference]	1 [Reference]	1 [Reference]
Female	1.18 (1.13-1.24)	1.16 (1.11-1.22)	1.06 (1.00-1.13)	0.99 (0.93-1.06)
Race/ethnicity				
White	1 [Reference]	1 [Reference]	1 [Reference]	1 [Reference]
Asian	0.64 (0.56-0.73)	0.71 (0.61-0.81)	0.62 (0.50-0.75)	1.05 (0.85-1.28)
Black	0.69 (0.62-0.78)	0.75 (0.66-0.84)	0.55 (0.46-0.66)	0.64 (0.54-0.77)
Latinx	0.79 (0.74-0.84)	0.85 (0.80-0.91)	0.60 (0.54-0.66)	0.81 (0.73-0.90)
Native American	0.92 (0.72-1.19)	0.94 (0.73-1.21)	0.79 (0.55-1.14)	0.91 (0.63-1.32)
Other	1.07 (0.95-1.20)	1.12 (0.99-1.26)	0.68 (0.56-0.83)	0.91 (0.74-1.12)
Unknown	1.51 (1.40-1.63)	1.71 (1.59-1.85)	0.41 (0.34-0.50)	0.84 (0.68-1.03)
Insurance payer				
Traditional HMO	1 [Reference]	1 [Reference]	1 [Reference]	1 [Reference]
Deductible or coinsurance	1.50 (1.38-1.62)	1.49 (1.37-1.61)	1.08 (0.94-1.24)	1.20 (1.05-1.38)
High deductible	1.88 (1.72-2.06)	1.88 (1.72-2.06)	0.94 (0.79-1.13)	1.22 (1.02-1.47)
Medicaid	1.24 (1.08-1.43)	1.21 (1.05-1.40)	0.96 (0.74-1.23)	0.98 (0.76-1.27)
Medicare	2.11 (1.95-2.29)	1.49 (1.31-1.70)	3.67 (3.25-4.15)	1.37 (1.13-1.67)
Other	0.90 (0.77-1.05)	0.90 (0.77-1.05)	1.32 (1.06-1.65)	1.33 (1.07-1.66)
Duration of enrollment, y				
≤1	1 [Reference]	1 [Reference]	1 [Reference]	1 [Reference]
2-5	0.86 (0.81-0.92)	1.05 (0.97-1.12)	1.22 (1.06-1.40)	1.53 (1.32-1.77)
>5	0.90 (0.84-0.96)	1.06 (0.98-1.14)	2.12 (1.86-2.42)	1.61 (1.40-1.87)
Address changes in prior year				
0	1 [Reference]	1 [Reference]	1 [Reference]	1 [Reference]
1	1.07 (1.01-1.14)	1.12 (1.05-1.20)	0.83 (0.75-0.92)	1.00 (0.90-1.12)
≥2	1.20 (1.06-1.35)	1.24 (1.10-1.41)	0.70 (0.56-0.88)	0.80 (0.64-1.02)
Received medical financial assistance				
No	1 [Reference]	1 [Reference]	1 [Reference]	1 [Reference]
Yes	0.96 (0.85-1.08)	0.68 (0.60-0.77)	1.52 (1.34-1.72)	0.71 (0.63-0.81)
**Clinical**				
Medical conditions				
0	1 [Reference]	1 [Reference]	1 [Reference]	1 [Reference]
1	0.87 (0.82-0.93)	0.85 (0.79-0.90)	1.61 (1.40-1.85)	1.09 (0.95-1.26)
2	0.90 (0.84-0.97)	0.82 (0.76-0.88)	1.93 (1.68-2.22)	1.01 (0.87-1.17)
≥3	0.98 (0.93-1.03)	0.66 (0.61-0.71)	3.38 (3.01-3.80)	0.81 (0.71-0.93)
Mental or behavioral health conditions				
0	1 [Reference]	1 [Reference]	1 [Reference]	1 [Reference]
1	0.95 (0.88-1.02)	0.86 (0.80-0.93)	1.38 (1.25-1.52)	1.02 (0.92-1.13)
2	1.09 (1.01-1.17)	0.95 (0.88-1.02)	1.43 (1.30-1.57)	0.90 (0.82-0.99)
≥3	1.13 (1.05-1.20)	0.80 (0.74-0.86)	1.94 (1.79-2.10)	0.92 (0.84-1.01)
Substance use				
No	1 [Reference]	1 [Reference]	1 [Reference]	1 [Reference]
Yes	1.18 (1.05-1.33)	1.11 (0.98-1.27)	1.44 (1.24-1.67)	0.87 (0.74-1.03)
Text messages per y				
1.0-1.9	1 [Reference]	1 [Reference]	NA	NA
2.0-2.9	0.70 (0.64-0.77)	0.74 (0.67-0.80)	NA	NA
3.0-4.9	0.70 (0.64-0.75)	0.74 (0.68-0.80)	NA	NA
5.0-9.9	0.80 (0.74-0.86)	0.88 (0.81-0.95)	NA	NA
10.0-19.9	1.10 (1.02-1.19)	1.27 (1.17-1.38)	NA	NA
≥20	2.91 (2.69-3.15)	3.58 (3.28-3.91)	NA	NA
Opted out of IVR messages				
No	1 [Reference]	1 [Reference]	NA	NA
Yes	8.05 (7.23-8.95)	5.92 (5.29-6.61)	NA	NA
IVR messages per y				
1.0-1.9	NA	NA	1 [Reference]	1 [Reference]
2.0-2.9	NA	NA	1.64 (1.34-2.01)	1.52 (1.24-1.86)
3.0-4.9	NA	NA	3.39 (2.85-4.02)	2.88 (2.42-3.42)
5.0-9.9	NA	NA	6.99 (5.97-8.18)	5.58 (4.75-6.55)
10.0-19.9	NA	NA	14.00 (11.96-16.40)	11.11 (9.43-13.08)
≥20	NA	NA	55.61 (47.61-64.95)	49.84 (42.33-58.70)
Opted out of text messages				
No	NA	NA	1 [Reference]	1 [Reference]
Yes	NA	NA	5.01 (4.52-5.56)	4.07 (3.65-4.55)

Abbreviations: IVR, interactive voice response; NA, not applicable; OR, odds ratio.

## Discussion

In this cohort study, we assessed the proportion of adult members of an integrated health care system who opted out of receiving future automated text messages or IVR telephone calls over a 1-year period. The incidence of opting out over the 1-year period was low for in the overall cohort but increased in association with message volume, particularly among individuals receiving IVR messages. Individuals who opted out of receiving either type of message were substantially more likely to opt out of the other type during the study year. Older individuals were more likely to opt out of either communication channel, whereas other sociodemographic, clinical, or social variables such as sex and race/ethnicity were not consistently associated with opting out. We also observed a transient increase in opt-out requests after text message campaigns that were broadly informational rather than focused on individual health care needs.

Message fatigue has long been a concern for marketing and advertising campaigns outside health care.^7,21^ Commercial vendors have reported opt-out rates for text message campaigns that range from 2% to 5%,^22,23,24^ comparable to the rates we observed. Little previous research has assessed the aggregate volume of automated messages in clinical practice or demonstrated an association between message volume and message fatigue in clinical or public health settings.^25^ Existing research^7,26,27^ has primarily assessed the attitudes expressed by recipients in response to health messages rather than requests to discontinue those messages. These studies have shown that message fatigue may be an unintended consequence of public health messages about safe sex, obesity prevention, and smoking cessation.^26,27,28,29^ Message fatigue may also jeopardize the effectiveness of public health messages during the COVID-19 pandemic.^30^

The findings of this study should be replicated in other health care systems. If additional research is confirmatory, future qualitative and quantitative studies should explore the complex relationships between patient attitudes about automated messages, their preferences for communication channels and message frequency, and their decisions to continue or opt out of future messages. Our findings suggest that the association of patient age with these attitudes is particularly important because opt-out rates for both text and IVR messages increased as age increased despite accumulating medical and social risk factors and greater use of care.

Despite the low rates of message fatigue that we observed, health care systems should consider approaches to reduce this problem and optimize the effectiveness of their automated communication strategies.^2,3,4^ Incorporating patient preferences about the channel, frequency, and topics of communication from the system could ensure that individuals receive the content they desire through the channels they prefer at a frequency they would accept. Awareness of personal preferences could also inform a coordinated governance and prioritization process among multiple entities within the delivery system that generate IVR-, text-, or email-based message campaigns.^31^ Messages that are currently delivered separately could be bundled into a single communication that identifies all upcoming health care needs.^29^ Predictive models could direct reminder messages about upcoming appointments to individuals at greatest risk of missing those appointments. Research in KPCO and other settings has shown that patients who will likely miss appointments can be predicted with high accuracy using electronic health record variables.^12,13,32^ “Blast” messages about topics that are important to the delivery system but have limited personal relevance may have unintended consequences that should be considered before deployment.

### Limitations

This study has limitations. The generalizability of our findings is limited by the inclusion of adult members from a single integrated health care system and by restriction of the analysis to a single year. We assessed only text and IVR messages, although email messages, in-person telephone calls, and text or IVR messages from national Kaiser Permanente sources also contributed to total message volume and may have exacerbated message fatigue.

Additional limitations of the study include our inability to assess the number of individuals who experienced message fatigue but did not opt out; a survey would be necessary to assess these attitudes.^26,27^ Because the decision to opt out of text messages was associated with cessation of all further texts, whereas the decision to opt out of IVR messages was associated only with cessation of calls for that specific purpose, opt-out rates and patient characteristics may not be comparable between the 2 communication channels. We could not determine why individuals chose to opt out in response to a specific message. Information on member preferences for communication channels was not consistently available.

## Conclusions

In this cohort study, a small proportion of adult KPCO members demonstrated message fatigue through requests to discontinue text and IVR messages from the system. The prior volume of messages received was associated with message fatigue. Recognition of this unintended consequence of automated text and IVR communication may promote message delivery strategies that improve patient-centeredness while preserving the effectiveness of these important tools.

## Notes

Supplement. **eTable 1.** Characteristics of Adult Members of Kaiser Permanente Colorado Who Received Vs Did Not Receive Automated Messages **eTable 2.** Characteristics of Adult Members of Kaiser Permanente Colorado With Different Profiles of Automated Messages **eTable 3.** Bivariate and Multivariate Risk Factors for Opt Out of Text and IVR Message Among Individuals With One or More In-Person Visits (“Active” Users)
