# Supplementary material for: Factors Associated With Opting Out of Automated Text and Telephone Messages Among Adult Members of an Integrated Health Care System
Source: JAMA Netw Open. 2021 Mar 26;4(3):e213479. doi: 10.1001/jamanetworkopen.2021.3479 (PMC7998073; doi:10.1001/jamanetworkopen.2021.3479)
Supplement: Supplement. — eTable 1. Characteristics of Adult Members of Kaiser Permanente Colorado Who Received Vs Did Not Receive Automated Messages eTable 2. Characteristics of Adult Members of Kaiser Permanente Colorado With Different Profiles of Automated Messages eTable 3. Bivariate and Multivariate Risk Factors for Opt Out of Text and IVR Message Among Individuals With One or More In-Person Visits (“Active” Users) [file jamanetwopen-e213479-s001.pdf]

Supplementary Online Content

Steiner JF, Zeng C, Comer AC, et al. Factors associated with opting out of automated text and telephone messages among adult members of an integrated health care system. *JAMA Netw Open*. 2021;4(3):e213479. doi:10.1001/jamanetworkopen.2021.3479

eTable 1. Characteristics of Adult Members of Kaiser Permanente Colorado Who Received Vs Did Not Receive Automated Messages
eTable 2. Characteristics of Adult Members of Kaiser Permanente Colorado With Different Profiles of Automated Messages
eTable 3. Bivariate and Multivariate Risk Factors for Opt Out of Text and IVR Messages Among Individuals with One or More In-Person Visits (“Active” Users)

This supplementary material has been provided by the authors to give readers additional information about their work.

© 2021 Steiner JF et al. *JAMA Network Open*.

eTable 1. Characteristics of Adult Members of Kaiser Permanente Colorado Who Received vs Did Not Receive Automated Messages				
**Characteristics**	**All members N =612,780**	**Did not receive message N =184,538**	**Received text messages or IVR calls N =428,242**	**P value**
**Sociodemographic characteristics**				
Age				<0.001
18-34	165,649 (27.0)	79,194 (42.9)	86,455 (20.2)	
35-54	205,392 (33.5)	68,650 (37.2)	136,742 (31.9)	
55-64	108,104 (17.6)	20,208 (11.0)	87,896 (20.5)	
65-79	105,732 (17.3)	13,070 (7.1)	92,662 (21.6)	
80 or older	27,903 (4.6)	3,416 (1.9)	24,487 (5.7)	
Sex				<0.001
Female	324,619 (53.0)	69,168 (37.5)	255,451 (59.7)	
Male	288,161 (47.0)	115,370 (62.5)	172,791 (40.3)	
Race/Ethnicity				<0.001
White	361,581 (59.0)	77,011 (41.7)	284,570 (66.5)	
Black	24,479 (4.0)	5,536 (3.0)	18,943 (4.4)	
Latinx	91,253 (14.9)	26,087 (14.1)	65,166 (15.2)	
Asian	20,132 (3.3)	5,401 (2.9)	14,731 (3.4)	
Native American	4,600 (0.8)	1,474 (0.8)	3,126 (0.7)	
Other	25,018 (4.1)	11,336 (6.1)	13,682 (3.2)	
Unknown	85,717 (14.0)	57,693 (31.3)	28,024 (6.5)	
Insurance payer				<0.001
Deductible/Coinsurance	256,602 (41.9)	94,027 (51.0)	162,575 (38.0)	
High deductible	84,303 (13.8)	31,434 (17.0)	52,869 (12.3)	
Traditional HMO	77,920 (12.7)	23,328 (12.6)	54,592 (12.7)	
Medicaid	25,779 (4.2)	10,099 (5.5)	15,680 (3.7)	
Medicare	142,803 (23.3)	16,522 (9.0)	126,281 (29.5)	

© 2021 Steiner JF et al. *JAMA Network Open*.

Other	25,373 (4.1)	9,128 (4.9)	16,245 (3.8)	
Duration of enrollment				<0.001
1 year or less	99,790 (16.3)	51,536 (27.9)	48,254 (11.3)	
2-5 years	222,572 (36.3)	76,867 (41.7)	145,705 (34.0)	
More than 5 years	290,418 (47.4)	56,135 (30.4)	234,283 (54.7)	

© 2021 Steiner JF et al. *JAMA Network Open*.

eTable 1. Characteristics of Adult Members of Kaiser Permanente Colorado who Received vs Did Not Receive Automated Messages (continued)				
**Characteristics**	**All members<br>N =612,780**	**Did not receive message<br>N =184,538**	**Received text messages or IVR calls<br>N =428,242**	**P value**
Number of address changes in prior year				<0.001
0	529,530 (86.4)	160,289 (86.9)	369,241 (86.2)	
1	69,826 (11.4)	21,420 (11.6)	48,406 (11.3)	
2 or more	13,424 (2.2)	2,829 (1.5)	10,595 (2.5)	
Received medical financial assistance				<0.001
No	597,143 (97.4)	183,296 (99.3)	413,847 (96.6)	
Yes	15,637 (2.6)	1,242 (0.7)	14,395 (3.4)	
**Clinical characteristics**				
Medical conditions				<0.001
0	203,571 (33.2)	105,385 (57.1)	98,186 (22.9)	
1	123,871 (20.2)	35,452 (19.2)	88,419 (20.6)	
2	84,410 (13.8)	17,891 (9.7)	66,519 (15.5)	
3 or more	200,928 (32.8)	25,810 (14.0)	175,118 (40.9)	
Mental-behavioral health conditions				<0.001
0	476,682 (77.8)	173,379 (94.0)	303,303 (70.8)	
1	46,542 (7.6)	5,474 (3.0)	41,068 (9.6)	
2	44,219 (7.2)	3,214 (1.7)	41,005 (9.6)	
3 or more	45,337 (7.4)	2,471 (1.3)	42,866 (10.0)	
Substance abuse				<0.001
No	600,376 (98.0)	182,990 (99.2)	417,386 (97.5)	
Yes	12,404 (2.0)	1,548 (0.8)	10,856 (2.5)	
**Utilization characteristics**				
Primary care visits in prior year				<0.001
0	253,908 (41.4)	153,191 (83.0)	100,717 (23.5)	
1	153,015 (25.0)	21,906 (11.9)	131,109 (30.6)	
2	88,094 (14.4)	6,254 (3.4)	81,840 (19.1)	
3 or more	117,763 (19.2)	3,187 (1.7)	114,576 (26.8)	

© 2021 Steiner JF et al. *JAMA Network Open*.

eTable 1. Characteristics of Adult Members of Kaiser Permanente Colorado who Received vs Did Not Receive Automated Messages (continued)

Characteristics	All members N =612,780	Did not receive message N =184,538	Received text messages or IVR calls N =428,242	*P* value
Specialty care visits in prior year				<0.001
0	380,844 (62.2)	176,917 (95.9)	203,927 (47.6)	
1	90,930 (14.8)	5,467 (3.0)	85,463 (20.0)	
2	47,126 (7.7)	1,134 (0.6)	45,992 (10.7)	
3 or more	93,880 (15.3)	1,020 (0.6)	92,860 (21.7)	
ED visits in prior year				<0.001
0	544,242 (88.8)	172,805 (93.6)	371,437 (86.7)	
1	44,935 (7.3)	7,923 (4.3)	37,012 (8.6)	
2 or more	23,603 (3.9)	3,810 (2.1)	19,793 (4.6)	
Hospitalizations in prior year				<0.001
0	584,145 (95.3)	182,009 (98.6)	402,136 (93.9)	
1	21,286 (3.5)	2,000 (1.1)	19,286 (4.5)	
2 or more	7,349 (1.2)	529 (0.3)	6,820 (1.6)	
Number of text messages per year				<0.001
0	252,418 (41.2)	184,538 (100.0)	67,880 (15.9)	
1.0-1.9	41,114 (6.7)	0 (0.0)	41,114 (9.6)	
2.0-2.9	55,938 (9.1)	0 (0.0)	55,938 (13.1)	
3.0-4.9	74,460 (12.2)	0 (0.0)	74,460 (17.4)	
5.0-9.9	103,322 (16.9)	0 (0.0)	103,322 (24.1)	
10.0-19.9	61,238 (10.0)	0 (0.0)	61,238 (14.3)	
20 or more	24,290 (4.0)	0 (0.0)	24,290 (5.7)	
Number of IVR calls per year				<0.001
0	322,374 (52.6)	184,538 (100.0)	137,836 (32.2)	
1.0-1.9	75,864 (12.4)	0 (0.0)	75,864 (17.7)	
2.0-2.9	49,903 (8.1)	0 (0.0)	49,903 (11.7)	

© 2021 Steiner JF et al. *JAMA Network Open*.

3.0-4.9	58,143 (9.5)	0 (0.0)	58,143 (13.6)	
5.0-9.9	61,722 (10.1)	0 (0.0)	61,722 (14.4)	
10.0-19.9	32,294 (5.3)	0 (0.0)	32,294 (7.5)	
20 or more	12,480 (2.0)	0 (0.0)	12,480 (2.9)	

Abbreviations: IVR, interactive voice response (telephone); HMO, health maintenance organization; ED, emergency department.

© 2021 Steiner JF et al. *JAMA Network Open*.

eTable 2. Characteristics of Adult Members of Kaiser Permanente Colorado With Different Profiles of Automated Messages

Characteristics	All members who received messages N =428,242	Text messages only N =137,836	IVR calls only N =67,880	Both Text and IVR messages N =222,526	*P* value
**Sociodemographic characteristics**					
Age					
18-34	86,455 (20.2)	44,667 (32.4)	4,961 (7.3)	36,827 (16.5)	<0.001
35-54	136,742 (31.9)	50,630 (36.7)	13,166 (19.4)	72,946 (32.8)	
55-64	87,896 (20.5)	24,945 (18.1)	14,334 (21.1)	48,617 (21.8)	
65-79	92,662 (21.6)	15,022 (10.9)	23,918 (35.2)	53,722 (24.1)	
80 or older	24,487 (5.7)	2,572 (1.9)	11,501 (16.9)	10,414 (4.7)	
Sex					
Female	255,451 (59.7)	76,227 (55.3)	41,021 (60.4)	138,203 (62.1)	<0.001
Male	172,791 (40.3)	61,609 (44.7)	26,859 (39.6)	84,323 (37.9)	
Race/Ethnicity					
White	284,570 (66.5)	82,237 (59.7)	46,699 (68.8)	155,634 (69.9)	<0.001
Black	18,943 (4.4)	5,996 (4.4)	2,526 (3.7)	10,421 (4.7)	
Latinx	65,166 (15.2)	24,551 (17.8)	7,819 (11.5)	32,796 (14.7)	
Asian	14,731 (3.4)	5,423 (3.9)	1,969 (2.9)	7,339 (3.3)	
Native American	3,126 (0.7)	951 (0.7)	496 (0.7)	1,679 (0.8)	
Other	13,682 (3.2)	5,033 (3.7)	2,170 (3.2)	6,479 (2.9)	
Unknown	28,024 (6.5)	13,645 (9.9)	6,201 (9.1)	8,178 (3.7)	
Insurance					
Deductible/Coinsurance	162,575 (38.0)	67,722 (49.1)	16,043 (23.6)	78,810 (35.4)	<0.001
High deductible	52,869 (12.3)	24,104 (17.5)	5,512 (8.1)	23,253 (10.4)	
Traditional HMO	54,592 (12.7)	16,681 (12.1)	5,313 (7.8)	32,598 (14.6)	
Medicaid	15,680 (3.7)	5,236 (3.8)	1,946 (2.9)	8,498 (3.8)	
Medicare	126,281 (29.5)	19,103 (13.9)	36,963 (54.5)	70,215 (31.6)	
Other	16,245 (3.8)	4,990 (3.6)	2,103 (3.1)	9,152 (4.1)	
Enrollment categories					

© 2021 Steiner JF et al. *JAMA Network Open*.

1 year or less	48,254 (11.3)	20,233 (14.7)	6,036 (8.9)	21,985 (9.9)	<0.001
2-5 years	145,705 (34.0)	58,985 (42.8)	15,141 (22.3)	71,579 (32.2)	
More than 5 years	234,283 (54.7)	58,618 (42.5)	46,703 (68.8)	128,962 (58.0)	

© 2021 Steiner JF et al. *JAMA Network Open*.

eTable 2. Characteristics of Adult Members of Kaiser Permanente Colorado With Different Profiles of Automated Messages (continued)

Characteristics	All members who received messages N =428,242	Text messages only N =137,836	IVR calls only N =67,880	Both Text and IVR messages N =222,526	*P* value
Number of address changes in prior year					
0	369,241 (86.2)	115,981 (84.1)	61,866 (91.1)	191,394 (86.0)	<0.001
1	48,406 (11.3)	18,298 (13.3)	5,032 (7.4)	25,076 (11.3)	
2 or more	10,595 (2.5)	3,557 (2.6)	982 (1.4)	6,056 (2.7)	
Received medical financial assistance					
No	413,847 (96.6)	135,517 (98.3)	65,301 (96.2)	213,029 (95.7)	<0.001
Yes	14,395 (3.4)	2,319 (1.7)	2,579 (3.8)	9,497 (4.3)	
**Clinical characteristics**					
Medical conditions					
0	98,186 (22.9)	47,408 (34.4)	12,292 (18.1)	38,486 (17.3)	<0.001
1	88,419 (20.6)	34,121 (24.8)	10,485 (15.4)	43,813 (19.7)	
2	66,519 (15.5)	21,562 (15.6)	8,733 (12.9)	36,224 (16.3)	
3 or more	175,118 (40.9)	34,745 (25.2)	36,370 (53.6)	104,003 (46.7)	
Mental-behavioral health conditions					
0	303,303 (70.8)	108,421 (78.7)	49,995 (73.7)	144,887 (65.1)	<0.001
1	41,068 (9.6)	11,568 (8.4)	5,927 (8.7)	23,573 (10.6)	
2	41,005 (9.6)	9,715 (7.0)	6,421 (9.5)	24,869 (11.2)	
3 or more	42,866 (10.0)	8,132 (5.9)	5,537 (8.2)	29,197 (13.1)	
Substance abuse					
No	417,386 (97.5)	135,622 (98.4)	66,510 (98.0)	215,254 (96.7)	<0.001
Yes	10,856 (2.5)	2,214 (1.6)	1,370 (2.0)	7,272 (3.3)	
**Utilization characteristics**					
Primary care visits in prior year					
0	100,717 (23.5)	47,582 (34.5)	16,758 (24.7)	36,377 (16.3)	<0.001
1	131,109 (30.6)	47,529 (34.5)	20,048 (29.5)	63,532 (28.6)	
2	81,840 (19.1)	22,957 (16.7)	11,997 (17.7)	46,886 (21.1)	

© 2021 Steiner JF et al. *JAMA Network Open*.

3 or more	114,576 (26.8)	19,768 (14.3)	19,077 (28.1)	75,731 (34.0)	

© 2021 Steiner JF et al. *JAMA Network Open*.

eTable 2. Characteristics of Adult Members of Kaiser Permanente Colorado With Different Profiles of Automated Messages (continued)

Characteristics	All members who received messages<br>N =428,242	Text messages only<br>N =137,836	IVR calls only<br>N =67,880	Both Text and IVR messages<br>N =222,526	*P* value
Specialty care visits in prior year					
0	203,927 (47.6)	113,171 (82.1)	31,725 (46.7)	59,031 (26.5)	<0.001
1	85,463 (20.0)	15,863 (11.5)	12,617 (18.6)	56,983 (25.6)	
2	45,992 (10.7)	4,703 (3.4)	7,055 (10.4)	34,234 (15.4)	
3 or more	92,860 (21.7)	4,099 (3.0)	16,483 (24.3)	72,278 (32.5)	
ED visits in prior year					
0	371,437 (86.7)	124,963 (90.7)	58,515 (86.2)	187,959 (84.5)	<0.001
1	37,012 (8.6)	8,632 (6.3)	6,135 (9.0)	22,245 (10.0)	
2 or more	19,793 (4.6)	4,241 (3.1)	3,230 (4.8)	12,322 (5.5)	
Hospitalizations in prior year					
0	402,136 (93.9)	133,772 (97.1)	62,823 (92.6)	205,541 (92.4)	<0.001
1	19,286 (4.5)	3,355 (2.4)	3,655 (5.4)	12,276 (5.5)	
2 or more	6,820 (1.6)	709 (0.5)	1,402 (2.1)	4,709 (2.1)	
Number of text message per year					
0	67,880 (15.9)	0 (0.0)	67,880 (100.0)	0 (0.0)	<0.001
1.0-1.9	41,114 (9.6)	24,874 (18.0)	0 (0.0)	16,240 (7.3)	
2.0-2.9	55,938 (13.1)	39,292 (28.5)	0 (0.0)	16,646 (7.5)	
3.0-4.9	74,460 (17.4)	33,965 (24.6)	0 (0.0)	40,495 (18.2)	
5.0-9.9	103,322 (24.1)	27,664 (20.1)	0 (0.0)	75,658 (34.0)	
10.0-19.9	61,238 (14.3)	9,077 (6.6)	0 (0.0)	52,161 (23.4)	
>=20.0	24,290 (5.7)	2,964 (2.2)	0 (0.0)	21,326 (9.6)	
Number of IVR calls per year					
0	137,836 (32.2)	137,836 (100.0)	0 (0.0)	0 (0.0)	<0.001
1.0-1.9	75,864 (17.7)	0 (0.0)	12,542 (18.5)	63,322 (28.5)	
2.0-2.9	49,903 (11.7)	0 (0.0)	10,000 (14.7)	39,903 (17.9)	
3.0-4.9	58,143 (13.6)	0 (0.0)	12,816 (18.9)	45,327 (20.4)	

© 2021 Steiner JF et al. *JAMA Network Open*.

5.0-9.9	61,722 (14.4)	0 (0.0)	17,557 (25.9)	44,165 (19.8)	
10.0-19.9	32,294 (7.5)	0 (0.0)	10,728 (15.8)	21,566 (9.7)	
>=20.0	12,480 (2.9)	0 (0.0)	4,237 (6.2)	8,243 (3.7)	

Abbreviations: IVR, interactive voice response (telephone); HMO, health maintenance organization; ED, emergency department.

© 2021 Steiner JF et al. *JAMA Network Open*.

eTable 3. Bivariate and Multivariate Risk Factors of Opt Out of Text and IVR Message Among Individuals With One or More In-Person Visits ("Active" Users)

Characteristics	Opted out of text messages		Opted out of IVR calls	
**Sociodemographic characteristics**	**Unadjusted OR (95% CI)**	**Adjusted OR (95% CI)**	**Unadjusted OR (95% CI)**	**Adjusted OR (95% CI)**
Age				
18-34	1.0 (ref)	1.0 (ref)	1.0 (ref)	1.0 (ref)
35-54	0.87 (0.81, 0.94)	0.95 (0.88, 1.03)	0.91 (0.79, 1.05)	0.96 (0.83, 1.11)
55-64	1.44 (1.33, 1.55)	1.57 (1.45, 1.70)	1.47 (1.28, 1.69)	1.35 (1.16, 1.57)
65-79	1.70 (1.59, 1.83)	1.61 (1.40, 1.86)	3.38 (2.99, 3.81)	2.17 (1.76, 2.67)
80 or older	1.74 (1.55, 1.95)	1.62 (1.36, 1.92)	5.06 (4.43, 5.77)	2.75 (2.20, 3.42)
Sex				
Male	1.0 (ref)	1.0 (ref)	1.0 (ref)	1.0 (ref)
Female	1.21 (1.16, 1.28)	1.17 (1.11, 1.23)	1.08 (1.01, 1.15)	1.01 (0.94, 1.08)
Race/ethnicity				
White	1.0 (ref)	1.0 (ref)	1.0 (ref)	1.0 (ref)
Asian	0.66 (0.57, 0.76)	0.76 (0.66, 0.89)	0.63 (0.52, 0.77)	1.05 (0.86, 1.29)
Black	0.69 (0.61, 0.79)	0.76 (0.67, 0.86)	0.55 (0.46, 0.66)	0.64 (0.53, 0.77)
Latinx	0.76 (0.71, 0.82)	0.84 (0.78, 0.90)	0.59 (0.54, 0.66)	0.80 (0.72, 0.89)
Native American	0.87 (0.65, 1.16)	0.88 (0.66, 1.18)	0.85 (0.59, 1.22)	0.94 (0.65, 1.37)
Other	0.92 (0.80, 1.06)	1.01 (0.87, 1.17)	0.75 (0.62, 0.92)	0.93 (0.76, 1.14)
Unknown	1.08 (0.96, 1.21)	1.28 (1.14, 1.45)	0.60 (0.49, 0.74)	0.99 (0.80, 1.23)
Insurance payer				
Traditional HMO	1.0 (ref)	1.0 (ref)	1.0 (ref)	1.0 (ref)
Deductible/Coinsurance	1.39 (1.28, 1.52)	1.41 (1.29, 1.54)	1.10 (0.96, 1.26)	1.19 (1.04, 1.37)
High deductible	1.66 (1.50, 1.84)	1.72 (1.56, 1.91)	0.98 (0.82, 1.18)	1.22 (1.01, 1.47)
Medicaid	1.13 (0.97, 1.32)	1.08 (0.92, 1.27)	0.98 (0.76, 1.27)	1.00 (0.77, 1.30)
Medicare	2.17 (1.99, 2.36)	1.50 (1.30, 1.73)	3.62 (3.20, 4.10)	1.36 (1.12, 1.65)
Other	0.85 (0.72, 1.02)	0.85 (0.72, 1.02)	1.34 (1.07, 1.67)	1.33 (1.06, 1.67)
Duration of enrollment				
1 year or less	1.0 (ref)	1.0 (ref)	1.0 (ref)	1.0 (ref)
2-5 years	0.91 (0.84, 0.98)	1.10 (1.01, 1.19)	1.10 (0.95, 1.27)	1.44 (1.24, 1.67)
More than 5 years	1.02 (0.95, 1.10)	1.13 (1.04, 1.23)	1.90 (1.66, 2.17)	1.50 (1.30, 1.74)
Number of address changes in prior year				
0	1.0 (ref)	1.0 (ref)	1.0 (ref)	1.0 (ref)
1	1.08 (1.00, 1.16)	1.15 (1.07, 1.24)	0.84 (0.75, 0.93)	1.01 (0.91, 1.13)

© 2021 Steiner JF et al. *JAMA Network Open*.

2 or more	1.29 (1.14, 1.47)	1.36 (1.19, 1.55)	0.68 (0.54, 0.85)	0.78 (0.62, 0.99)
Received medical financial assistance				
No	1.0 (ref)	1.0 (ref)	1.0 (ref)	1.0 (ref)
Yes	1.02 (0.91, 1.16)	0.68 (0.6, 0.77)	1.43 (1.26, 1.62)	0.70 (0.61, 0.80)

© 2021 Steiner JF et al. *JAMA Network Open*.

eTable 3. Bivariate and Multivariate Risk Factors of Opt Out of Text and IVR Message Among Individuals With One or More In-Person Visits (“Active” Users) (continued)				
**Characteristics**	**Opted out of text messages**		**Opted out of IVR calls**	
**Sociodemographic characteristics**	**Unadjusted OR (95% CI)**	**Adjusted OR (95% CI)**	**Unadjusted OR (95% CI)**	**Adjusted OR (95% CI)**
**Clinical characteristics**				
Medical conditions				
0	1.0 (ref)	1.0 (ref)	1.0 (ref)	1.0 (ref)
1	1.02 (0.95, 1.10)	0.92 (0.85, 0.99)	1.47 (1.28, 1.70)	1.06 (0.92, 1.23)
2	1.05 (0.97, 1.14)	0.86 (0.79, 0.94)	1.72 (1.49, 1.98)	0.96 (0.83, 1.12)
3 or more	1.20 (1.12, 1.28)	0.69 (0.63, 0.75)	2.98 (2.65, 3.36)	0.78 (0.68, 0.89)
Mental-behavioral health conditions				
0	1.0 (ref)	1.0 (ref)	1.0 (ref)	1.0 (ref)
1	1.06 (0.98, 1.15)	0.95 (0.88, 1.03)	1.30 (1.18, 1.43)	1.01 (0.91, 1.12)
2	1.22 (1.14, 1.32)	1.03 (0.95, 1.11)	1.34 (1.22, 1.48)	0.89 (0.81, 0.98)
3 or more	1.28 (1.19, 1.37)	0.86 (0.79, 0.93)	1.82 (1.68, 1.97)	0.90 (0.82, 0.99)
Substance abuse				
No	1.0 (ref)	1.0 (ref)	1.0 (ref)	1.0 (ref)
Yes	1.28 (1.13, 1.46)	1.13 (0.99, 1.29)	1.39 (1.20, 1.61)	0.89 (0.75, 1.05)
Number of text messages per year			NA	NA
1.0-1.9	1.0 (ref)	1.0 (ref)		
2.0-2.9	0.78 (0.69, 0.87)	0.79 (0.70, 0.88)		
3.0-4.9	0.76 (0.68, 0.85)	0.74 (0.66, 0.82)		
5.0-9.9	0.89 (0.81, 0.98)	0.85 (0.77, 0.94)		
10.0-19.9	1.27 (1.15, 1.40)	1.24 (1.12, 1.38)		
20 or more	3.60 (3.26, 3.97)	3.71 (3.33, 4.13)		
Opted out of IVR calls			NA	NA
No	1.0 (ref)	1.0 (ref)		
Yes	8.79 (7.88, 9.80)	6.04 (5.39, 6.76)		
Number of IVR calls per year	NA	NA		
1.0-1.9			1.0 (ref)	1.0 (ref)
2.0-2.9			1.57 (1.28, 1.93)	1.45 (1.18, 1.78)
3.0-4.9			3.10 (2.60, 3.70)	2.64 (2.21, 3.15)
5.0-9.9			6.30 (5.35, 7.42)	5.06 (4.28, 5.97)
10.0-19.9			12.62 (10.73, 14.85)	10.11 (8.55, 11.95)
20 or more			51.18 (43.61, 60.07)	46.26 (39.11, 54.71)

© 2021 Steiner JF et al. *JAMA Network Open*.

Opted out of text messages	NA	NA		
No			1.0 (ref)	1.0 (ref)
Yes			5.00 (4.50, 5.55)	4.00 (3.57, 4.47)

© 2021 Steiner JF et al. *JAMA Network Open*.
